# Impact of disease duration on forefoot involvement in rheumatoid arthritis remission: clinical, ultrasound, and radiographic insights

**DOI:** 10.1186/s41927-026-00649-5

**Published:** 2026-05-02

**Authors:** Rebeca Bueno Fermoso, Jose Luis Maté-Muñoz, Carmen Martínez Rincón, Juan Miguel López González, Rubén Sánchez-Gómez, Maria Luz González Fernández

**Affiliations:** 1 Faculty of Nursing, Physiotherapy and Podiatry, Complutense University of Madrid, Madrid, 28040 Spain; 2 Hospital Universitario General de Villalba, Collado Villalba, 28400 Spain

**Keywords:** Rheumatoid arthritis, Forefoot, Ultrasound, Remission, Disease duration

## Abstract

**Background:**

Rheumatoid arthritis (RA) frequently affects the forefoot, causing pain and deformity even in remission. Subclinical inflammation may persist and vary with disease duration. This study aimed to compare clinical, ultrasound, and radiographic features of the forefoot in RA remission with metatarsalgia according to disease duration (< 10 vs. ≥ 10 years).

**Methods:**

Cross-sectional study including 84 RA patients in remission (DAS28 < 2.6) with metatarsalgia: 33 with < 10 and 51 with ≥ 10 years of disease duration. Clinical, biomechanical, ultrasound (B-mode and Power Doppler (PD), and radiographic variables were assessed by blinded evaluators.

**Results:**

The ≥ 10-year group showed greater structural burden. Total synovitis was similar between groups, more frequent at central metatarsophalangeal joints (MTPs) (particularly MTP2 in < 10 years), and PD was rare (6%). The overall pattern was consistent with early MTP1/5 involvement and progressive structural accumulation at MTP2–4, with higher odds of erosions (OR 3.5–5.0), joint space narrowing (JSN; OR 2.8–4.4), and subluxations (MTP2 OR 5.31; MTP3 OR 2.50) in the ≥ 10-year group.

**Conclusions:**

In RA remission with metatarsalgia, our findings are consistent with early structural involvement at MTP1/MTP5 and more frequent B-mode synovitis at central joints, with progressive structural burden at MTP2–4 as disease duration increases. PD positivity was uncommon (6%), whereas ultrasound remained essential to detect synovitis. Our findings support systematic assessment of all MTP joints (MTP1–5), with particular attention to MTP1/MTP5 in earlier disease and careful structural evaluation of central rays in longer disease duration. As a cross-sectional study, temporal or causal sequences cannot be inferred.

## Key points

In RA remission, forefoot pain, B-mode synovitis, and structural damage can persist.

Structural involvement appears early at MTP1/5 and, with longer disease duration, progresses predominantly at MTP2–4.

Residual synovitis is more frequent at central MTPs (MTP2–4); PD signal is infrequent but clinically relevant, so Doppler ultrasound is needed to detect residual vascular activity.

## Introduction

Rheumatoid arthritis (RA) is characterized by persistent synovitis [1], which leads to structural damage and functional limitation [2–4], including involvement of the foot, a key region for mobility and quality of life.

The foot is affected in a high proportion of patients throughout the disease course, with a prevalence of up to 90% [5–8]. Within the foot, the forefoot is the most frequently involved region [9], supporting its inclusion in specific structural damage scores [10].

Over time, RA is associated with progressive accumulation of structural damage and foot deformities [5, 11, 12], which has been linked to progressive functional limitation [3].

Moreover, the distribution of damage is not uniform, showing inter-individual variation [13], as well as differences by foot region [13, 14] and disease duration [14, 15], suggesting distinct phenotypes of involvement.

Controlling inflammation and preventing structural damage remain central goals in RA management. Clinical remission is the main therapeutic target; however, the most widely used activity indices, such as DAS28 [16], exclude foot joints, possibly underestimating residual activity in this location [16–18]. Indeed, patients in remission have been reported to exhibit foot pain or swelling [18], reinforcing the need for specific foot assessment even in apparent clinical remission. In this context, ultrasound, given its greater sensitivity to detect synovitis, has proven useful in foot evaluation, although some reduced protocols tend to underrepresent the forefoot [19, 20]. Additionally, ultrasound studies have demonstrated subclinical ankle involvement in RA, supporting comprehensive foot/forefoot assessment even during remission [21].

In this setting, evidence integrating clinical, radiographic, and ultrasound assessment of the forefoot in RA patients in remission is still scarce. A 10-year disease duration cut-off has previously been used as an operational criterion to differentiate clinical, biomechanical, and structural characteristics of the rheumatoid foot [22, 23], since many deformities require years to consolidate and become clinically evident [14].

Objective: The objective was to evaluate, according to disease duration (< 10 vs. ≥ 10 years), the morphofunctional, clinical, ultrasound, and radiographic characteristics of the forefoot in RA patients in remission with metatarsalgia, and to analyze the joint distribution of involvement.

## Materials and methods

### Study design and setting

A cross-sectional study was conducted between January 2023 and June 2024 at the Rheumatic Foot Unit, University Podiatry Clinic, Faculty of Nursing, Physiotherapy and Podiatry, Complutense University of Madrid. Patients were eligible if their rheumatologist had confirmed a diagnosis of RA [24], they presented with metatarsal pain, and were in clinical remission according to DAS28 (< 2.6) [16]. Participants were stratified by disease duration, using a 10-year cut-off previously applied in similar studies [22, 23].

All analyses followed STROBE guidelines for cross-sectional studies.

Given the cross-sectional design, all analyses reflect associations and should not be interpreted as temporal changes.

### Participants

Patients were consecutively recruited, and only those fulfilling the inclusion criteria and providing written informed consent were enrolled.

**Inclusion criteria**:

- Diagnosis of RA according to ACR/EULAR 2010 classification criteria [24] and clinical remission (DAS28 < 2.6) [16], both confirmed by the rheumatologist.
- Presence of metatarsal pain at the time of evaluation.
- Age ≥ 18 years.
- Ability to undergo clinical assessment and comply with data collection.
- Availability of weight-bearing dorsoplantar radiographs obtained within the previous 3 months.

**Exclusion criteria**:

- Previous foot surgery.
- Other rheumatic or neurodegenerative diseases, or diabetes mellitus.
- Orthopedic treatment or intra-articular injections within the previous 3 months.
- Active tumors or prior history of foot masses.

### Sample size

Sample size was determined by feasibility, including all consecutive patients evaluated during the study period, consistent with previous studies applying the 10-year cut-off [22, 23]. A total of 84 patients were enrolled: 33 with < 10 years and 51 with ≥ 10 years of disease duration.

### Bias control

Consecutive sampling was applied to reduce selection bias. Clinical examinations were performed by a podiatrist specialized in biomechanics (> 15 years of experience), blinded to imaging data. Ultrasound assessments were conducted by a second investigator with > 30 years of experience in rheumatic patients, blinded to clinical and radiographic information. Radiographs were anonymized and independently evaluated by a third assessor (> 30 years of experience), blinded to both clinical and ultrasound findings.

Interobserver reliability was tested in a random subsample of 15 radiographs. The kappa coefficient for erosions and joint space narrowing (JSN), as well as the ICC for the structural index score (SIS), were all > 0.80, indicating excellent agreement.

### Variables and data sources

Sociodemographic variables (age, sex, BMI), disease duration, pain type and intensity (mechanical and/or inflammatory; VAS scale [25]), and current pharmacological treatment were obtained through a structured clinical interview and review of rheumatology records.

Biomechanical variables were assessed by the first examiner and included:

- First metatarsophalangeal (MTP) joint mobility, measured in non–weight-bearing with a goniometer [26]. Limited motion was defined as < 40° or a positive functional hallux limitus test [27], and rigidity as < 10° [28].
- Subtalar joint mobility, assessed in prone, non–weight-bearing using a goniometer. Limitation was defined as inversion < 18–20° [29].
- Ankle dorsiflexion (Silfverskiöld test), assessed in supine position with the foot in neutral alignment. Limitation was defined as < 90° with the knee extended and < 10–12° with the knee flexed [30].
- Foot type, classified using the Foot Posture Index (FPI) [31]. Feet were categorized as pronated (pronated + highly pronated), supinated (supinated + highly supinated), or neutral.
- Lesser toe deformities [32], recorded as present/absent in the sagittal plane without specifying type. Flexibility was assessed using the Kelikian push-up test [33] and categorized as flexible, semi-flexible, or rigid.
- Fifth ray deformity (Taylor’s bunion), recorded as present/absent [34].
- Hallux valgus, graded using the Manchester scale [35].
- Forefoot Structural Index Score (SIS) [2], evaluating deformities, dislocations, hallux valgus, and fifth MTP exostosis; total range 0–12.

Ultrasound examinations were performed by the second examiner using a Samsung HS50 ultrasound system (Samsung Medison Co., Ltd., Seoul, South Korea) equipped with a high-frequency linear array transducer (model LA3-14AD) operating at 3–14 MHz, specifically designed for musculoskeletal imaging. The musculoskeletal/small-parts preset was applied.

The examinations were performed in B-mode and power Doppler mode, with frequency settings between 10 and 14 MHz for superficial structures and a Doppler frequency of 5–7 MHz. The pulse repetition frequency (PRF) was set between 500 and 800 Hz, and gain and depth were adjusted individually to optimize visualization of joint structures and vascular signals. For PD, gain was increased to the background-noise threshold and then reduced slightly below it, using a low wall filter, a small Doppler box centered on the synovium, and minimal probe pressure to avoid flow compression, to enhance reproducibility. Image acquisition and lesion definitions followed OMERACT 2019 ultrasound recommendations for inflammatory arthritis. B-mode synovitis and power Doppler (PD) were evaluated in MTP1–5 [36]. Synovitis was defined as present when B-mode ≥ 1, and PD activity as present when PD ≥ 1 (both dichotomized at ≥ 1 according to OMERACT 2019). We recorded the total number of MTP joints with B-mode synovitis (range 0–5), the total number with PD signal (range 0–5), and the specific MTP joint(s) involved. Examinations were performed with the patient seated, foot supported, and the probe placed longitudinally over the dorsal aspect of each MTP joint. All scans were performed with the same machine, transducer and preset. To indicate measurement reliability, 15 images were independently reviewed by another operator; Cohen’s kappa was 0.80.

Radiographic variables were assessed on weight-bearing dorsoplantar radiographs [37]. Erosions (present/absent) and joint space narrowing (JSN; present/absent) were recorded for MTP1–5 and the hallux interphalangeal joint, according to the Simple Erosion Narrowing Score (SENS) scoring system [10]. The total foot score (0–12) was calculated as erosions (0–6) + JSN (0–6). Because dislocations often accompany JSN, they were analyzed separately in the MTP joints [38].

### Missing data

All participants completed the clinical, ultrasound, and radiographic assessments, with no missing data.

### Statistical analysis

Quantitative variables were reported as mean ± SD (if normally distributed) or median [IQR] (if non-normal), and categorical variables as n (%). Normality was assessed with the Shapiro–Wilk test and homogeneity of variances with Levene’s test. Between-group comparisons (< 10 vs. ≥ 10 years) used independent-samples t tests or Mann–Whitney U tests as appropriate; categorical data were compared using χ² or Fisher’s exact tests. For joint-level outcomes, odds ratios (ORs) with 95% confidence intervals (CIs) were calculated. All tests were two-sided with α = 0.05. Analyses were conducted using IBM SPSS Statistics, version 28.0 (IBM Corp.). Interobserver reliability for ultrasound and radiographic readings (presence/absence outcomes) was quantified using Cohen’s kappa (κ) in SPSS v28.0.

### Ethical considerations

The study was conducted in accordance with the principles of the Declaration of Helsinki (Fortaleza revision, 2013) and was approved by the Ethics Committee of Hospital Clínico San Carlos (protocol no. 21/719-E). All participants received detailed information about the study objectives and procedures and provided written informed consent.

## Results

A total of 84 patients with RA in clinical remission and metatarsalgia were included: 33 with disease duration < 10 years (RA < 10) and 51 with ≥ 10 years (RA ≥ 10). The mean disease duration was 5.6 ± 2.1 years in RA < 10 and 27.6 ± 12.2 years in RA ≥ 10.

### Baseline characteristics

Baseline characteristics are summarized in Table 1. Most participants were women in both groups. Patients with RA ≥ 10 years were significantly older, whereas BMI did not differ between groups. Regarding treatment, NSAID use and calcium supplementation were more frequent in RA ≥ 10. In terms of health habits, smoking was more prevalent in RA < 10, while sedentary lifestyle predominated in RA ≥ 10.

**Table 1:** Baseline characteristics of the study population

Characteristic	RA < 10 years (*n* = 33)	RA ≥ 10 years (*n* = 51)	*p*-value
Demographics			
Female sex, n (%)	29 (87.9)	49 (96.1)	0.216
Age, years, mean ± SD	58.0 ± 10.7	64.3 ± 12.4	0.020
BMI, kg/m², mean ± SD	26.4 ± 4.2	25.1 ± 3.9	0.144
Treatment, n (%)			
Steroids	12 (36.4)	28 (54.9)	0.097
csDMARDs	22 (66.7)	41 (80.4)	0.156
Biologics	10 (30.3)	23 (45.1)	0.175
NSAIDs	11 (33.3)	28 (54.9)	0.001
Paracetamol	2 (6.1)	10 (19.6)	0.094
Antiplatelet agents	0 (0)	4 (7.8)	0.183
Calcium supplements	12 (36.4)	32 (62.7)	0.024
Vitamin D supplements	14 (42.4)	30 (58.8)	0.16
Comorbidities, n (%)			
Hypertension	12 (36.4)	11 (21.6)	0.321
Hypercholesterolemia	11 (33.3)	20 (39.2)	0.339
Hypothyroidism	3 (9.1)	14 (27.5)	0.074
Health habits, n (%)			
Smoking	14 (42.1)	4 (7.9)	0.001
Sedentary lifestyle	12 (37.5)	30 (58.0)	< 0.001

### Pain characteristics

Pain intensity (VAS) did not differ between groups (7.12 ± 1.88 vs. 7.39 ± 2.17; *p* = 0.214). However, pain type distribution differed significantly (*p* < 0.001): mechanical pain predominated in RA ≥ 10, whereas inflammatory pain was more frequent in RA < 10, while mixed pain was the most common type in both groups. Results are presented in Table 2.

**Table 2:** Pain characteristics of the study population

Variable	RA < 10 years (*n* = 33)	RA ≥ 10 years (*n* = 51)	*p*-value	95% CI
Pain VAS, mean ± SD	7.12 (1.88)	7.39 (2.17)	0.214	-1.18 / 0.64
Pain type, n (%)			< 0.001	
Mechanical	8 (24.2)	21 (41.2)		
Inflammatory	6 (18.2)	1 (2.0)		
Mixed	17 (51.5)	26 (51.0)		
Other	2 (6.1)	3 (5.9)		

Values are expressed as mean ± SD or n (%). VAS: Visual Analog Scale. RA < 10: rheumatoid arthritis < 10 years of disease duration; RA ≥ 10: rheumatoid arthritis ≥ 10 years of disease duration

### Clinical examination

#### Joint mobility, digital deformities and foot posture

No significant between-group differences were observed in ankle dorsiflexion or overall first MTP mobility. By contrast, subtalar joint limitation was significantly more frequent in RA ≥ 10 (*p* < 0.001). Rigid limitation of the first MTP joint was more commonly observed at RA ≥ 10 (*p* = 0.039).

Digital deformities were frequent in both groups without overall significance (*p* = 0.146). However, subluxations/dislocations (*p* = 0.004), Taylor’s bunion (*p* = 0.032), and rigid deformities on the Kelikian test (*p* < 0.001) were significantly more frequently observed in RA ≥ 10. Hallux deformities were also more prevalent in RA ≥ 10 (*p* = 0.007), while the distribution of Manchester grades did not differ significantly between groups (*p* = 0.161).

The Foot Posture Index (FPI) showed a predominance of supinated feet in RA < 10 and neutral feet in RA ≥ 10, but the difference was not significant. The mean forefoot Structural Index Score (SIS) was significantly higher in RA ≥ 10 (see Table 3).

**Table 3:** Joint mobility, digital deformities and foot posture

Variable	RA < 10 years (*n* = 33)	RA ≥ 10 years (*n* = 51)	*p*-value	95% CI
Limited ankle dorsiflexion	24 (72.7)	38 (74.5)	0.911	
1st MTP joint – limited	16 (48.5)	33 (64.7)		
1st MTP joint – rigid	1 (3.0)	6 (11.8)	0.039	
Positive hallux limitus functional test	24 (72.7)	40 (78.4)	0.549	
STJ inversion limitation	5 (15.2)	25 (49.0)	< 0.001	
Digital deformities	21 (63.6)	38 (74.5)	0.146	
Subluxations/dislocations	16 (48.4)	40 (78.4)	0.004	
Taylor’s bunion	8 (24.2)	26 (53.1)	0.032	
Kelikian test			< 0.001	
Correctable	18 (54.5)	10 (19.6)		
Partially correctable	11 (33.3)	24 (47.1)		
Rigid	4 (12.1)	17 (33.3)		
Hallux deformities	25 (75.8)	44 (86.3)	0.007	
Manchester scale			0.161	
Grade 1	15 (45.5)	19 (37.2)		
Grade 2	12 (36.4)	15 (29.4)		
Grade 3	4 (12.1)	5 (9.8)		
Grade 4	2 (6.1)	12 (23.5)		
Foot Posture Index (FPI)			0.111	
Neutral foot	7 (21.2)	20 (39.2)		
Pronated	4 (12.1)	12 (23.5)		
Supinated	22 (66.7)	19 (37.3)		
Forefoot SIS, mean ± SD	5.06 (3.61)	7.47 (3.23)	0.002	-3.91 / -0.90

Values are expressed as n (%) or mean ± SD. RA < 10: rheumatoid arthritis < 10 years of disease duration; RA ≥ 10: rheumatoid arthritis ≥ 10 years of disease duration. HV: hallux valgus; FPI: Foot Posture Index; SIS: Structural Index Score. TPA: ankle joint; MTP: metatarsophalangeal joint; HLF: hallux limitus functional test; STJ: subtalar joint

### Ultrasound and radiographic evaluation

The mean number of MTP joints with synovitis did not differ between groups (2.61 ± 1.32 vs. 2.43 ± 1.84; *p* = 0.307). Doppler signal was rare (< 10%) and evenly distributed across joints. Patients with RA ≥ 10 years showed significantly greater structural damage in the MTP joints, including more erosions, joint space narrowing (JSN), and subluxations/dislocations. The total SENS score was also significantly higher in RA ≥ 10 (Table 4).

**Table 4:** Ultrasound and radiographic evaluation

Variable	RA < 10 (*n* = 33)	RA ≥ 10 (*n* = 51)	*p*-value	95% CI
Ultrasound				
Total synovitis (MTP 1–5), mean ± SD	2.61 (1.32)	2.43 (1.84)	0.307	-0.51 / 0.86
Doppler-positive MTP (1–5), mean ± SD	0.06 (0.24)	0.10 (0.41)	–	–
Radiographic				
Erosions (SENS), mean ± SD	1.85 (1.50)	3.10 (1.69)	< 0.001	-1.97 / -0.53
JSN (SENS), mean ± SD	2.79 (1.96)	4.20 (1.92)	< 0.001	-2.27 / -0.55
Total SENS, mean ± SD	4.58 (3.05)	7.00 (2.96)	< 0.001	-3.76 / -1.09
Erosions (MTP 1–5), mean ± SD	1.78 (1.36)	2.82 (1.50)	0.002	-1.68 / -0.39
JSN (MTP 1–5), mean ± SD	2.36 (1.79)	3.60 (1.75)	0.002	-2.04 / -0.45
Subluxation/dislocation (MTP 1–5), mean ± SD	1.84 (1.98)	2.76 (1.71)	0.027	-1.73 / -0.10

Values are expressed as mean ± SD. RA < 10: rheumatoid arthritis < 10 years of disease duration; RA ≥ 10: rheumatoid arthritis ≥ 10 years of disease duration; JSN: joint space narrowing; SENS: Simple Erosion Narrowing Score (simplified Sharp–van der Heijde method); MTP: metatarsophalangeal joint

In adjusted regression models, both age and disease duration were significantly associated with structural damage outcomes (total SENS, erosions, JSN, and SIS). However, the effect was more pronounced for disease duration. By contrast, neither age nor disease duration was associated with the number of joints with synovitis (*p* = 0.301).

#### Joint-level analysis of synovitis and structural damage

When each MTP joint was analyzed individually, synovitis was significantly more frequent in MTP2 among RA < 10 patients (OR 0.28, *p* = 0.027). No other joint-level differences reached statistical significance.

**Fig. 1:**
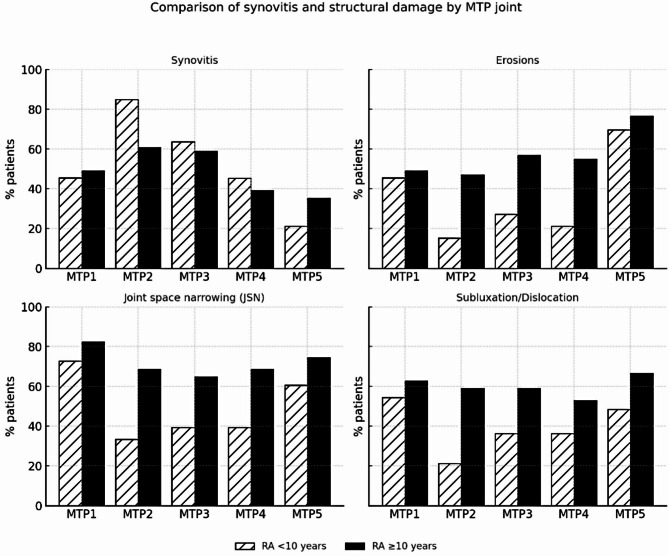
Prevalence of metatarsophalangeal involvement in RA remission according to disease duration. Bar plots depict the percentage of affected joints in RA < 10 and RA ≥ 10, stratified by lesion type: (**A**) synovitis, (**B**) erosions, (**C**) joint space narrowing (JSN), and (**D**) subluxations/dislocations

**Fig. 2:**
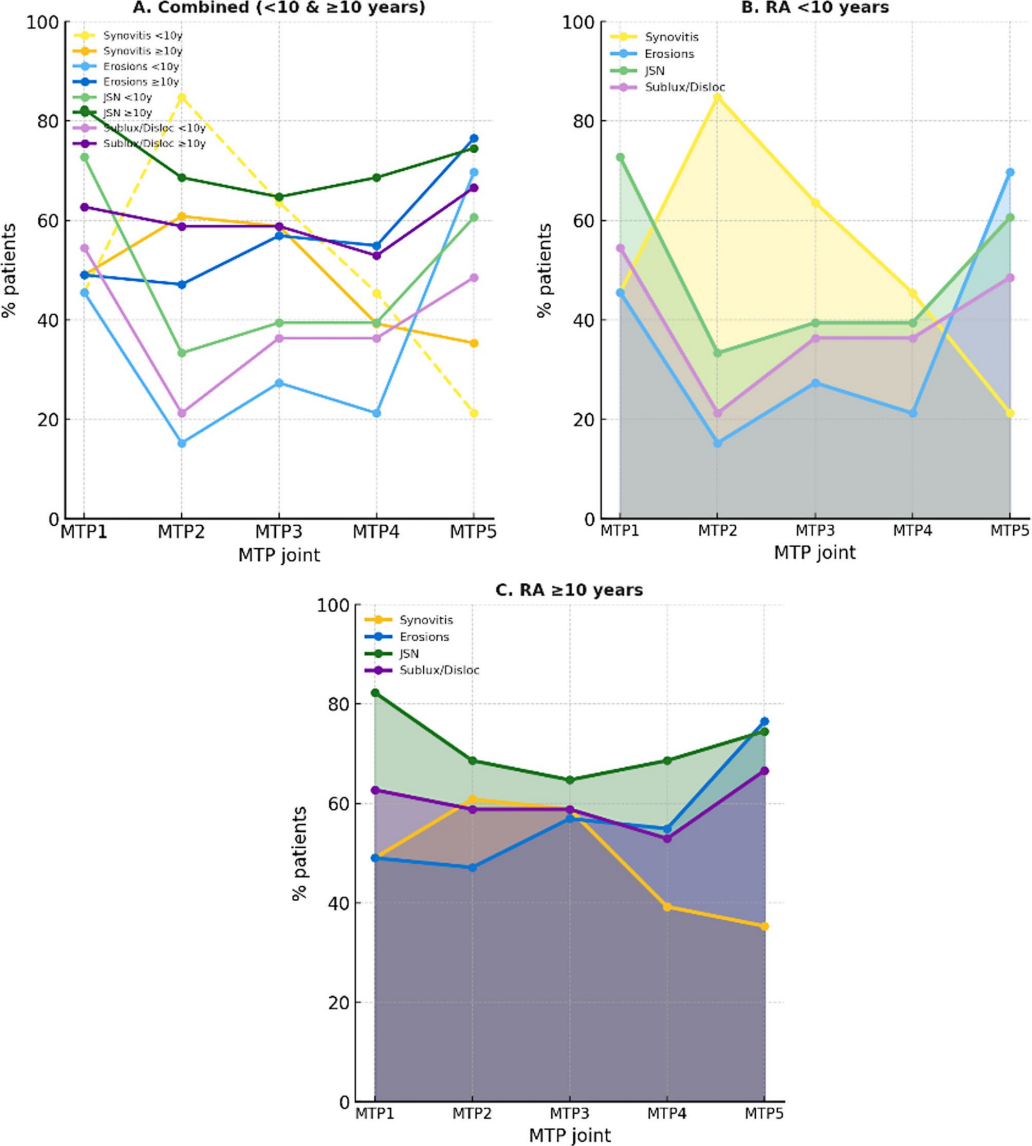
Prevalence of metatarsophalangeal involvement in RA remission according to disease duration. Bar plots depict the percentage of affected joints in RA < 10 and RA ≥ 10, stratified by lesion type and disease duration: (yellow) synovitis, (blue) erosions, (green) joint space narrowing (JSN), and (purple)subluxations/dislocations

For radiographic outcomes, RA ≥ 10 patients had significantly higher frequencies of erosions (MTP2–4, OR 3.5–5.0), JSN (MTP2–4, OR 2.8–4.4), and subluxations/dislocations (MTP2–3, OR 2.5–5.3). Both groups showed early involvement of MTP1 and MTP5, with MTP5 consistently exhibiting high frequencies of erosions and JSN (Table 5; Figs. 1 and 2).

**Table 5:** Localization of synovitis and radiographic damage in the forefoot (MTP joints 1–5)

Joint / Variable	RA < 10 (*n* = 33)	RA ≥ 10 (*n* = 51)	*p*-value	OR (95% CI)
Synovitis				
MTP1	15 (45.5)	25 (49.0)	0.749	1.15 (0.48–2.78)
MTP2	28 (84.8)	31 (60.8)	0.027	0.28 (0.09–0.84)
MTP3	21 (63.6)	30 (58.8)	0.659	0.82 (0.33–2.01)
MTP4	15 (45.4)	20 (39.2)	0.571	0.77 (0.32–1.88)
MTP5	7 (21.2)	18 (35.3)	0.168	2.03 (0.74–5.58)
Erosions				
MTP1	15 (45.5%)	25 (49.0%)	0.410	1.15 (0.48–2.78)
MTP2	5 (15.2%)	24 (47.1%)	0.003	4.98 (1.66–14.94)
MTP3	9 (27.3%)	29 (56.9%)	0.008	3.52 (1.37–9.05)
MTP4	7 (21.2%)	28 (54.9%)	0.002	4.52 (1.66–12.30)
MTP5	23 (69.7%)	39 (76.5%)	0.490	1.41 (0.53–3.78)
JSN				
MTP1	24 (72.7)	42 (82.3)	0.291	1.75 (0.61–5.01)
MTP2	11 (33.3)	35 (68.6)	0.003	4.38 (1.72–11.14)
MTP3	13 (39.4)	33 (64.7)	0.023	2.82 (1.14–6.97)
MTP4	13 (39.4)	35 (68.6)	0.008	3.37 (1.35–8.40)
MTP5	20 (60.6)	38 (74.5)	0.168	1.90 (0.74–4.87)
Subluxation/Dislocation				
MTP1	18 (54.5)	32 (62.7)	0.455	1.40 (0.58–3.42)
MTP2	7 (21.2)	30 (58.8)	0.001	5.31 (1.94–14.48)
MTP3	12 (36.3)	30 (58.8)	0.044	2.50 (1.01–6.16)
MTP4	12 (36.3)	27 (52.9)	0.137	1.97 (0.80–4.83)
MTP5	16 (48.5)	34 (66.6)	0.097	2.12 (0.87–5.21)

Values are expressed as n (%). RA < 10: rheumatoid arthritis with < 10 years of disease duration; RA ≥ 10: rheumatoid arthritis with ≥ 10 years of disease duration; MTP: metatarsophalangeal joint; JSN: joint space narrowing. Odds ratios (OR) were calculated using RA < 10 as the reference (SPSS Crosstabs, Risk statistics). OR > 1 indicates greater frequency in RA ≥ 10; OR < 1 indicates greater frequency in RA < 10. Unadjusted ORs (reference group RA < 10); no between-group test for PD due to very low counts

## Discussion

RA is a systemic disease with a major impact on the foot even during apparent clinical remission. As observed in our cohort, patients in remission continued to present with pain and ultrasound-detected synovitis [6, 16, 18]. Our results are in line with ultrasound evidence of subclinical ankle involvement in RA, which supports systematic foot/forefoot assessment even during remission [21]. Moreover, structural damage may progress even in the absence of active inflammation [39]. Given its progressive nature, clinical and structural manifestations may differ by disease duration [12].

In this context, the present study provides evidence of differences in clinical, biomechanical, and structural features between patients with different disease durations, using a 10-year cut-off previously reported in the literature [21, 22]. The integrated evaluation enabled the identification of distinct involvement patterns not captured by global scoring systems. To our knowledge, no prior studies have jointly analyzed clinical, ultrasound, and radiographic findings at the joint level in RA patients in remission with metatarsalgia.

In our predominantly female cohort, consistent with RA epidemiology [40], pain intensity was comparable between groups, with mixed pain being the most frequent type. This finding reflects the complexity of pain in inflammatory diseases [41], where inflammatory and mechanical mechanisms may coexist even in remission. A trend toward a more inflammatory profile was observed in patients with < 10 years of disease, whereas mechanical pain predominated in those with ≥ 10 years, consistent with between-group differences in the relative contribution of inflammatory and structural components rather than temporal change [1, 41]. The greater use of NSAIDs in the ≥ 10-year group may reflect symptomatic prescription rather than active inflammation. This observation should be interpreted cautiously, as NSAID use may also be influenced by clinician preferences, concomitant musculoskeletal comorbidities, and treatment accessibility [42]. Given the cross-sectional design, these observations are associative.

Patients with ≥ 10 years of disease presented more clinical deformities, greater joint rigidity, and higher radiographic burden (erosions, joint space narrowing, and subluxations), in line with studies documenting structural progression in RA [11, 22, 23, 43]. Among these deformities, lesser toe deformities were more frequent and rigid in RA ≥ 10, as confirmed by a significantly higher SIS and more irreducible findings on the Kelikian test. Taylor’s bunion was also more prevalent in this group, supporting the notion that some alterations require years to consolidate and possibly corresponding to the triangular foot pattern described in the literature. In our cross-sectional comparison, these findings indicate higher structural burden in the longer-duration group and should not be interpreted as longitudinal progression.

Regarding hallux valgus, although gradual progression with disease duration is usually reported, we found a greater prevalence in our cohort than in the study by Reinoso-Cobo et al. [23], which included patients with hindfoot pain. The higher prevalence of hallux valgus in < 10 years in our series compared with the cohort of Reinoso-Cobo et al. [23] may reflect the inclusion of patients with metatarsal pain, suggesting a specific early forefoot involvement pattern. This indicates that not all deformities are late findings and may reflect distinct clinical phenotypes, as also noted by Matsumoto [14].

Subtalar joint limitation was more frequent in long-standing disease, consistent with more global foot involvement in advanced stages [14, 44]. By contrast, ankle dorsiflexion was limited in both groups, likely related to the clinical profile of our cohort centered on metatarsalgia [45], as gastrocnemius tightness is a known factor contributing to metatarsal pain. The first MTP joint was also more rigid in longer disease duration, consistent with greater structural burden in that group. These comparisons are associative and not temporal.

Total synovitis load (B-mode) was similar between groups, and Doppler signal was rare (5/84 patients; 7 MTP joints), with no association with disease duration, consistent with remission status and intensively treated cohorts [19]. Nevertheless, ultrasound remains useful for detecting residual activity [16], particularly since all patients reported pain. We observed greater synovitis in MTP2 in < 10 years, supporting preferential central-ray involvement in early phases, as also reported by Ma et al. [46].

Radiographically, patients with ≥ 10 years showed higher frequencies of erosions, JSN, and subluxations, as well as higher overall foot SENS scores, consistent with cumulative damage. In our cohort, MTP2–4 were more frequently affected in the longer-duration group, whereas MTP1 and MTP5 showed early involvement. The persistent vulnerability of the fifth ray, with erosions from early stages, aligns with previous descriptions [5, 14].

Overall, the pattern suggests that RA patients in clinical remission with metatarsal pain still present synovitis in the forefoot, albeit with limited Doppler activity.

Central-ray synovitis—particularly at MTP2—was more frequent in patients with shorter disease duration, whereas greater structural damage at the same central joints (MTP2–4) was observed in those with longer duration. In contrast, structural lesions at MTP1 and MTP5 were already common in the shorter-duration group, suggesting early vulnerability of the first and fifth rays to bony damage and deformities. Given the cross-sectional design, these observations should be further evaluated in longitudinal studies.

In global score analyses, disease duration showed a stronger association than age with both radiographic structural burden (SENS) and total ultrasound synovitis (B-mode), suggesting that disease progression, rather than aging, is the main driver of forefoot involvement. These associations indicate stronger links with disease duration than age, but causal inferences are not supported by the study design.

### Clinical implications and limitations

#### Clinical implications

These findings highlight the need for systematic evaluation of all MTP joints in RA patients in remission who present with metatarsalgia. Ultrasound remains essential to detect residual synovitis, and Power Doppler (PD) assessment should be included to identify vascular activity. Early involvement of MTP1 and MTP5 suggests that these peripheral rays may be initial sites of disease expression, whereas cumulative structural damage more often affects the central joints (MTP2–4) as disease duration increases. Accordingly, a routine forefoot ultrasound assessment (B-mode and PD) is advisable in painful remission, with early, priority evaluation of MTP1 and MTP5, both ultrasonographically and radiographically, and subsequent systematic assessment of the remaining MTP joints. Given the high prevalence and progressive nature of deformities, these should also be examined routinely during forefoot assessment. Nonetheless, the cross-sectional design warrants confirmation and refinement of this protocol in longitudinal studies.

#### Limitations

The main strength of this study lies in the integrated approach (clinical–ultrasound–radiographic) and joint-level analysis. Limitations include, first, the cross-sectional design precludes causal inference and prevents any statement about disease progression.

Second, although patients were referred from five hospitals, all belonged to a single autonomous region and were evaluated at one specialized podiatry clinic; this setting may introduce referral bias and slightly limit generalizability. Third, the absence of a comparator group without metatarsalgia restricts extrapolation to the entire remission population; however, this sampling enhances the clinical specificity of the “remission with forefoot pain” phenotype, allowing a more precise and meaningful description of this subgroup in clinical and podiatric terms. Fourth, unmeasured confounding, particularly treatment exposure and age-related biomechanical factors, cannot be excluded and may have influenced the observed associations. Variables such as sex or medication were not included in the multivariable analyses, so the results should be interpreted with caution.

## Conclusion

In RA patients in clinical remission with metatarsalgia, we observed persistent ultrasound-detected synovitis, predominantly in the central metatarsal joints, although PD signal was infrequent, as expected in remission. Importantly, this suggests that forefoot pain in remission may often reflect residual structural damage or biomechanical overload rather than ongoing inflammation, with Doppler positivity identifying the minority of patients with true inflammatory activity. In contrast, structural damage and deformities increased with longer disease duration, following a pattern characterized by early involvement of MTP1 and MTP5 and subsequent progression to the central joints (MTP2–4) in advanced stages. These findings underscore the need for systematic clinical, ultrasound (B-mode and PD), and radiographic assessment of all MTP joints, even in patients in apparent remission, to optimize structural outcomes and guide early preventive and therapeutic interventions. MTP1 and MTP5 may represent key targets for preventive strategies in early disease stages. Future longitudinal studies should confirm this sequential pattern and evaluate its predictive value for forefoot health. As a cross-sectional study, our findings reflect between-group associations and do not establish temporal or causal relationships.

## Data Availability

The datasets generated and/or analysed during the current study are available from the corresponding author on reasonable request.

## Abbreviations

ACR: American College of Rheumatology
bDMARDs: Biologic disease-modifying antirheumatic drugs
BMI: Body mass index
CI: Confidence interval
csDMARDs: Conventional synthetic disease-modifying antirheumatic drugs
DAS28: Disease Activity Score in 28 joints
DP: Dorsoplantar
EULAR: European Alliance of Associations for Rheumatology
FPI: Foot Posture Index
ICC: Intraclass correlation coefficient
IP: Interphalangeal
IQR: Interquartile range
JSN: Joint space narrowing
MTPs: Metatarsophalangeal joints
NSAIDs: Non-steroidal anti-inflammatory drugs
OR: Odds ratio
PD: Power Doppler
RA: Rheumatoid arthritis
SENS: Simple Erosion Narrowing Score (simplified Sharp–van der Heijde method)
SIS: Structural Index Score
SD: Standard deviation
STROBE: Strengthening the Reporting of Observational Studies in Epidemiology
VAS: Visual analogue scale
